# Correction: Rare earth metal (Sm)-doped NiMnO_3_ nanostructures for highly competent alkaline oxygen evolution reaction

**DOI:** 10.1039/d5na90073e

**Published:** 2025-10-29

**Authors:** S. Swathi, R. Yuvakkumar, G. Ravi, Abdullah G. Al-Sehemi, Dhayalan Velauthapillai

**Affiliations:** a Department of Physics, Alagappa University Karaikudi 630 003 Tamil Nadu India yuvakkumarr@alagappauniversity.ac.in; b Research Centre for Advanced Materials Science, King Khalid University Abha 61413 Saudi Arabia; c Department of Chemistry, King Khalid University Abha 61413 Saudi Arabia; d Faculty of Engineering and Science, Western Norway University of Applied Sciences Bergen 5063 Norway dhayalan.Velauthapillai@hvl.no

## Abstract

Correction for ‘Rare earth metal (Sm)-doped NiMnO_3_ nanostructures for highly competent alkaline oxygen evolution reaction’ by S. Swathi *et al.*, *Nanoscale Adv.*, 2022, **4**, 2501–2508, https://doi.org/10.1039/D2NA00022A.

The authors regret there is an apparent similarity in the XRD and Raman patterns of the Sm-doped NiMnO_3_ samples in Fig. 1A and B in the original article. The authors supplied the raw data and an independent expert verified the traces were different. The authors state the similarity in the two graphs is due to a processing error that occurred when they plotted the smoothed data and applied the smoothing to the wrong dataset. The authors have provided the graphs plotted with the original raw data without applying the smoothing function.

**Fig. 1:**
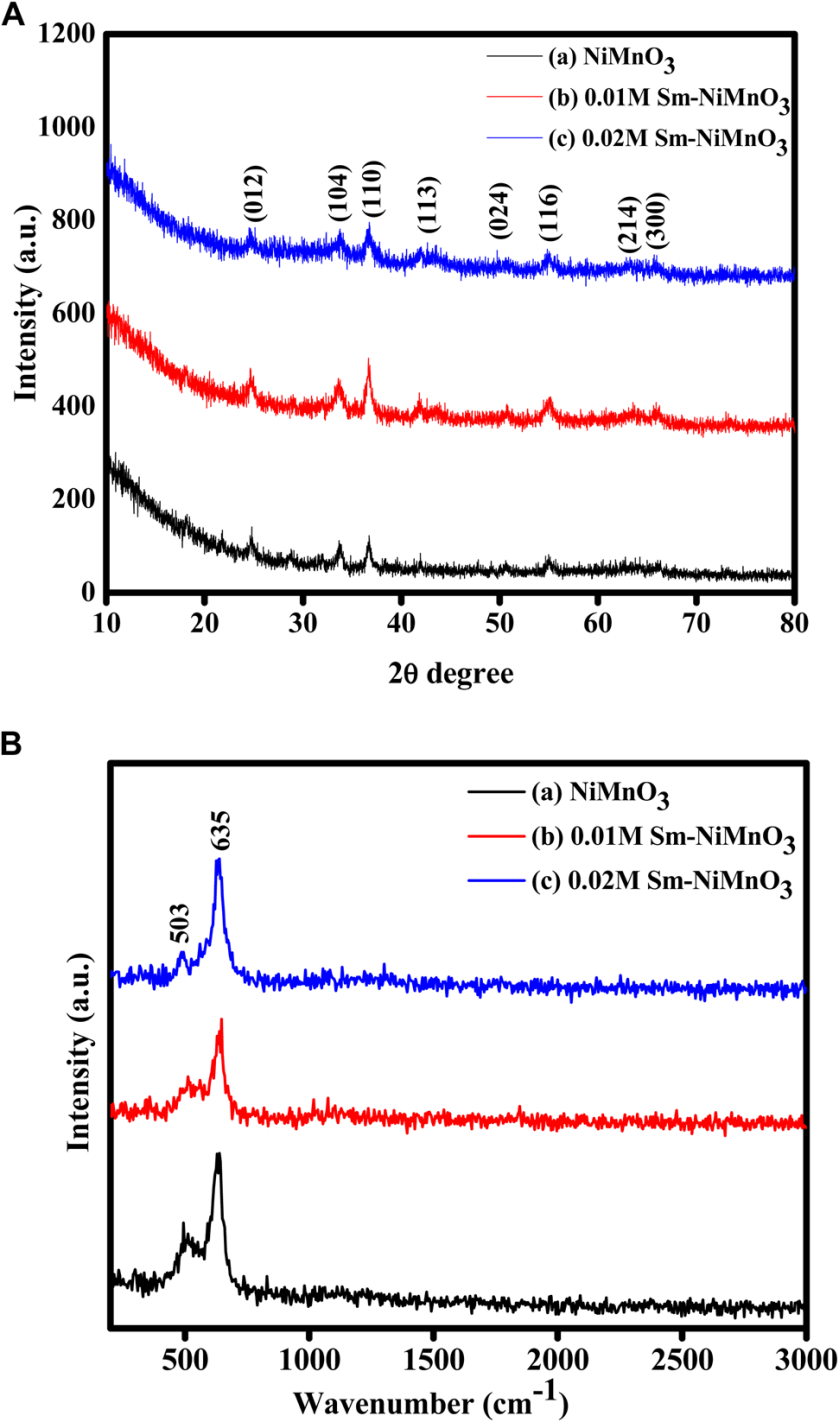
(A) XRD spectra of pristine NiMnO_3_, 0.01 M Sm-doped NiMnO_3_, and 0.02 M Sm-doped NiMnO_3_. (B) Raman spectra of pristine NiMnO_3_, 0.01 M Sm-doped NiMnO_3_, and 0.02 M Sm-doped NiMnO_3_.

The authors state the XRD and Raman characterization presented in Fig. 1A and B of this published paper is one of several characterization methods employed to analyze the samples and is of supportive nature. The unfortunate mistake made during selection of data smoothening process for plotting has no consequences in results, discussions, conclusions or outcomes of the published article.

The Royal Society of Chemistry apologises for these errors and any consequent inconvenience to authors and readers.

